# The Systematic Psychodynamic Multidimensional Organizational Assessment Questionnaire: Protocol for a Development and Validation Study

**DOI:** 10.2196/78530

**Published:** 2026-03-02

**Authors:** Anne-Maria Müller, Sophia Sachs, Yannik Rieder, Claas Lahmann

**Affiliations:** 1 Department of Psychosomatic Medicine and Psychotherapy, Faculty of Medicine, University of Freiburg, University Medical Center Freiburg, Hauptstrasse 8, Freiburg, 79104, Germany, 49 761-270 ext 68812

**Keywords:** questionnaire, organizational assessment, systems psychodynamics, psychodynamic theory, organizational health

## Abstract

**Background:**

Systems psychodynamics provide valuable insights into organizational development. However, to date, instruments that can reliably assess organizations based on systems psychodynamic theories are scarce. The Systematic Multidimensional Organizational Assessment (SyMOA) is a qualitative instrument that provides an in-depth systems psychodynamic analysis of organizational dynamics using a semistructured interview guide. To complement the method, a standardized, quantitative self-assessment questionnaire will be developed and validated.

**Objective:**

The aim of this study is to develop and psychometrically validate an instrument for assessing organizational dynamics based on the SyMOA diagnostic system. The questionnaire is intended to provide a scientifically grounded yet practical diagnostic tool applicable in both research and corporate practice. The findings aim to contribute to the advancement of systems psychodynamic theory and to serve as a foundation for evidence-based interventions in organizational change processes.

**Methods:**

The study follows a multistage development and validation process. First, the SyMOA instrument will be transformed into a questionnaire battery, and the items will be evaluated by experts (expert validity). The items will be tested through factor and item analyses by an online panel (n=150) and iteratively refined. Test-retest reliability will subsequently be assessed in a separate sample (n=150). Finally, factorial and discriminant validity will be examined in a larger validation sample (n=800).

**Results:**

As of February 2026, a first draft of 158 items has been developed based on dimension 1 of the SyMOA framework. The draft has undergone an expert review process with 2 experts in psychodynamics, who provided feedback on content validity and conceptual alignment. Approximately 20% of the items have been revised to improve clarity and theoretical precision. Data collection using a panel started in May 2025 and concluded in late August 2025, with iterative item analysis conducted thereafter.

**Conclusions:**

This study will evaluate the methodological robustness and usability of a questionnaire-based operationalization of systems psychodynamic organizational diagnostics. The anticipated findings are intended to guide further instrument development, normative calibration, and the responsible application of systems psychodynamic approaches in organizational research and practice.

## Introduction

### Background

The increasing complexity, volatility, and emotional demands of modern organizational life have heightened the need for diagnostic approaches that can access not only observable behaviors but also the latent, unconscious dynamics influencing individuals and systems. While conventional organizational diagnostics typically emphasize cognitive, behavioral, or structural dimensions, they often overlook the dynamics that drive behavior beneath the surface [1,2]. Systems psychodynamic diagnostics are a vital and underdeveloped avenue for capturing these phenomena in a scientifically grounded manner.

Rooted in the Tavistock tradition, the systems psychodynamic approach integrates psychoanalytic concepts with open systems theory to analyze how unconscious processes manifest within individuals, teams, and organizational cultures [2-5]. Key concepts include social defense mechanisms, unconscious conflicts, and the projection of anxiety onto systems or structures. These ideas provide a theoretical foundation for understanding phenomena such as resistance to change, dysfunctional leadership dynamics, or behaviors not adequately explained by surface-level assessments.

Despite its conceptual richness, the systems psychodynamic paradigm has historically relied on qualitative methodologies such as organizational role analysis [6], reflective team consultations [7,8], and projective methods such as metaphor elicitation and storytelling [9-11]. These tools offer deep insight but often lack psychometric validation—rendering them less suitable for large-scale organizational research.

On the one hand, organizational practitioners and researchers acknowledge the importance of unconscious dynamics; on the other hand, they lack scalable diagnostic instruments that can reliably quantify such processes while respecting the depth and contextual sensitivity of the systems psychodynamic framework. The challenge lies in designing diagnostics that do not reduce complex unconscious phenomena to simplistic metrics but, rather, operationalize key constructs with theoretical fidelity and psychometric validity.

This study protocol describes the development and validation of a novel psychometric instrument designed to assess unconscious dynamics in organizational contexts: the Systematic Multidimensional Organizational Assessment–Questionnaire (SyMOA-Q). Drawing on both psychodynamic constructs and empirical approaches, the aim is to create a self-assessment questionnaire that is theoretically grounded, methodologically rigorous, and practically useful for both researchers and organizational consultants.

### Prior Work

The SyMOA-Q builds on the authors’ previous work, the Systematic Multidimensional Organizational Assessment (SyMOA; C Lahmann et al, unpublished report), a qualitative systems psychodynamic diagnostic method that assesses organizational dynamics based on 3 dimensions (Table 1). The SyMOA was developed using a theory-to-research approach based on psychodynamic and organizational theories and was refined through an iterative process of theory development, empirical application, and theoretical refinement. It combines the logic of clinical diagnostics based on the *Operationalized Psychodynamic Diagnosis* [12] and organizational research. The SyMOA is a comprehensive and in-depth instrument that captures systemic and psychodynamic aspects within an organization that, to the best of our knowledge, no existing instrument has been able to map so far. A follow-up study testing the quality of the instrument, in particular the intercoder reliability and construct validity, is in preparation [13].

**Table 1.** Systematic Multidimensional Organizational Assessment dimensions.

Dimension	Description
Dimension 1: sociotechnical integration and the organization’s internal level of functioning	The first dimension describes the organization’s understanding of current and past challenges, including its resources and barriers to change. Additionally, this dimension delves deeper beneath the surface to examine the organization’s internal level of functioning. This includes how the organization as a system perceives itself and its environment; how it regulates itself both internally and externally in everyday situations as well as in critical moments; how internal and external communication functions; and how well it establishes bonds both internally among employees and externally with stakeholders such as customers, the market, and supply chains. Furthermore, this dimension captures the defense mechanisms typically used by the organization.
Dimension 2: internal relationship dynamics of the organization	The second dimension assesses the relationship dynamics between different groups within the organization. This includes relationship patterns in typical situations between individual employees, within teams, between teams, between management and stakeholders, and between management and employees.
Dimension 3: unconscious organizational conflicts	On the basis of psychodynamic theories of motivation, emotions, and unconscious conflicts, this dimension conceptualizes the internal conflicts within organizations. Typical conflicts include dependence vs autonomy, control vs subordination, intrinsic vs extrinsic organization-based self-worth conflicts, provision vs self-sufficiency, organizational exploitation vs prosocial engagement, market dominance, and identity conflicts. Conflicts are initially external, rooted in the early history of the company, but over time they become internalized and, thus, develop into unconscious internal conflicts. Conflict represents the clash of opposing positions, the collision of motives, desires, needs, values, and ideas. Conflicts between competing needs are a normal and everyday occurrence, such as between the need for self-determination in the workplace on the one hand and the desire for leadership and guidance on the other. If the underlying conflict is adequately resolved, individuals within the organization can shift flexibly between these needs depending on the situation. However, stress or dysfunction arises when the organization fails to develop an adequate strategy to resolve the fundamental conflict and persistently tries to rigidly implement one pole of these needs. These conflicts are repeatedly triggered by situational factors throughout the organization’s development.

### Research Objectives

Given the predominance of qualitative approaches in systems psychodynamic organizational research and the absence of validated quantitative instruments, this study seeks to develop and examine the psychometric properties of a questionnaire that operationalizes dimension 1 of the SyMOA framework. The objective is to establish an initial measurement model that captures core systems psychodynamic capacities—such as communication, attachment, awareness, and regulation—as perceived by organizational members. By providing a theoretically grounded and empirically testable tool, this study addresses a critical gap in the literature and lays the foundation for subsequent validation of the remaining SyMOA dimensions.

## Methods

### Aim, Design, and Setting

The qualitative methodology of the SyMOA will be converted into a standardized, quantitative self-assessment instrument. This study will first develop and validate a questionnaire for dimension 1 of the SyMOA diagnostics: sociotechnical integration and the organization’s internal level of functioning. The reliability and validity of the newly developed instrument, particularly factorial and discriminant validity, will be determined. A panel will be used for participant recruitment and data collection. The methodological approach of this study follows established best practices in psychometric instrument development and validation, as demonstrated in prior research across diverse applied fields such as health psychology, behavioral assessment, and attitudinal measurement [14-16]. These studies inform the multistage design adopted in this study, including pilot testing, reliability assessment, and factorial validation. The study was initially registered in the Freiburg Register for Clinical Studies (FRKS005727), which is not publicly accessible. It was later additionally registered in the German Clinical Trials Register (DRKS00039410) to ensure public access of the trial record.

### Study Procedure and Measures

**Figure 1.**
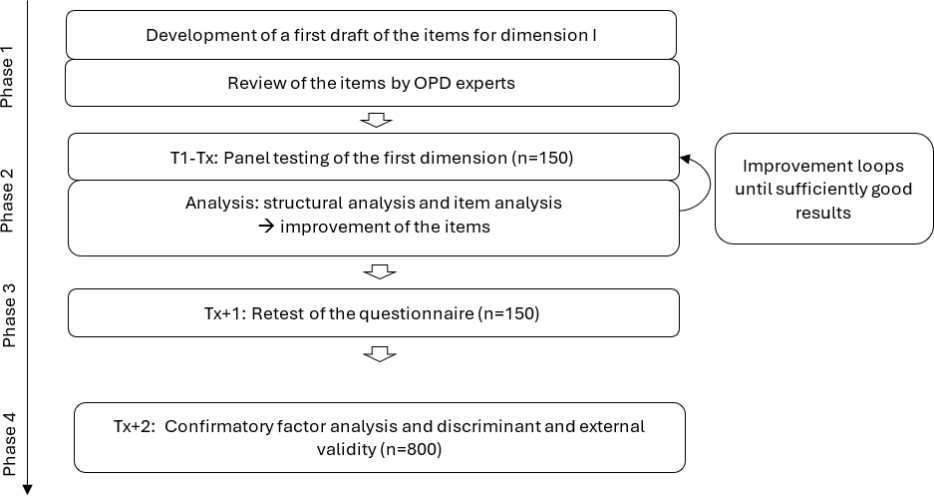
Study flow diagram. OPD: *Operationalized Psychodynamic Diagnosis*.

The study follows a multistage development and validation process, which is scheduled in 4 phases (Figure 1). However, although data collection spans several months, each participant is involved only briefly, completing the questionnaire once or, at most, twice.

First, the SyMOA scale for dimension 1 will be converted into a set of items and evaluated by 2 experts (expert validity). To this end, a preliminary set of items for the dimension will be created based on the qualitative SyMOA instrument. The aim is to have at least 80 items, which will be answered on a 5-point Likert scale. The items will be presented in German and mostly formulated negatively, with 20% formulated positively. In a second step, the items will be tested using structural and item-level analyses by an online panel sample (n=150) and refined iteratively based on their psychometric performance. Data collection will be conducted via LimeSurvey (LimeSurvey GmbH), a secure web-based survey platform. The development process will be completed if no further adjustment of the items is necessary.

As a third step, the retest reliability will be checked by sending the questionnaire to the same sample as that in the previous round (n=150) after 2 weeks. In a fourth step, a confirmatory factor analysis (CFA) will be carried out to check the fit of the theoretically assumed factor structure based on the SyMOA construct (sample of n=800). In addition, the discriminant validity of the questionnaires will be assessed. For this purpose, existing questionnaires used in clinical practice, such as the *Operationalisierte Psychodynamische Diagnostik–Strukturfragebogen* (OPD-SF) [17], will be used. To be able to assume that the surveyed constructs relate to the organization rather than the person completing the questionnaire, there should be a maximum of moderate correlations. For the evaluation of external validity, employee satisfaction (*Inventar zur Erfassung der Arbeitszufriedenheit* [18]) and work-related quality of life (World Health Organization Quality of Life Scale–Brief [19]) will also be administered in the last round. Although neither scale directly measures the assumed construct of organizational health dynamics, it can be assumed that there is a strong correlation between organizational health and the satisfaction and quality of life exhibited by employees. In addition, sociodemographic data such as age, gender, educational qualifications, professional experience, employment relationship, position or management function, sector, size of the company (eg, number of employees: 30-50 or 50-150), and size of the work area (eg, number of employees: 1-5, 5-10, or 10-20) will be evaluated in relation to the results.

For the development of the questionnaire for T1 to Tx (ie, from the start of data collection to the final item analysis round), as well as for the verification of the test-retest reliability for Tx+1 and the confirmatory sample for Tx+2, the recorded variables and their operationalization are listed in Table 2.

**Table 2.** Data collection with instruments and time point of measurement.

Time point of measurement and measured variable	Operationalization
T1^a^-Tx^b^—development of questionnaire based on dimension 1	
Quality of the items of the scale	Item analysis: item difficulty, item selectivity, item variance, and distribution
Reliability of the scale	Internal consistency of the scale
Tx+1^c^—retest reliability	
Retest reliability	Agreement of the results of Tx+1 with those of Tx
Tx+2^d^—CFA^e^ and validity	
Internal level of functioning of the organization	SyMOA-Q^f^ based on dimension 1 of the SyMOA^g^
Factorial validity of the scales	CFA
Sociodemographic data	Age, gender, educational qualifications, professional experience, employment relationship, position or management function, sector, size of the company (30-50, 50-150, 150-250, 250‐1000, or >1000), and size of work area (1-5, 5-10, 10-20, 20-50, and >50)
Discriminant validity via structural level of employees	OPD-SFK [17]^h^
Employee satisfaction as an external criterion	IAZ^i^ [18]
Work-related quality of life as an external criterion	WHOQOL-BREF [19]^j^

a T1 marks the start of data collection.

b Tx refers to the final round of phase 2 (completion of item analysis).

c Tx+1 indicates the retest reliability assessment (phase 3).

d Tx+2 refers to the confirmatory factor analysis and validity testing (phase 4).

e CFA: confirmatory factor analysis.

f SyMOA-Q: Systematic Multidimensional Organizational Assessment–Questionnaire.

g SyMOA: Systematic Multidimensional Organizational Assessment.

h OPD-SFK: *Operationalisierte Psychodynamische Diagnostik–Strukturfragebogen Kurz*.

i IAZ: Inventar zur Erfassung der Arbeitszufriedenheit.

j WHOQOL-BREF: World Health Organization Quality of Life Scale–Brief.

### Recruitment and Data Collection

The panel will be used for participant recruitment and data collection. Potential participants are registered users who meet the inclusion criteria. They must be aged ≥18 years, speak German, and be employed. Furthermore, the company with which they work must have at least 30 employees. Illiterate persons will be excluded. A message with the link to the study and the offer to participate will be sent to the database of potential participants until the required subsample of 150 people is reached for the first rounds. For the last round, a sample of 800 persons will be recruited.

Before the study, participants will receive written information about the procedure and any expected risks and side effects and the names of the people to contact. Informed consent will be obtained electronically in advance from the participants who agree to continue with the study.

### Data Analysis and Measures

#### Power

A sample size of 150 will be used for the iterative development of the questionnaire to check the retest reliability, internal consistency, and item analyses. This is methodologically justified as item analyses [20], internal consistency [21], and retest reliability [22] require between 100 and 200 people for a robust estimate.

For the CFA, the aim is to achieve a sample size of at least 800 following the rule of thumb by Bühner [23], with medium communalities (*h*^2^>0.50) and approximately 20 factors across all dimensions.

#### Statistical Analysis

The quantitative data will be analyzed using SPSS (IBM Corp), R (R Foundation for Statistical Computing), and JASP. The following statistical evaluations will be performed: a CFA (global fit using the chi-square test and goodness-of-fit indexes [root mean square error of approximation<0.05; Tucker-Lewis index and comparative fit index>0.9]), local fit (factor loadings>0.4), iterative item analyses (item distribution and variance, item difficulty [0.2-0.8], and discriminatory power [>0.3]), and verification of correlations and internal consistency of the scales (retest reliability [>0.7] and discriminant validity). The data from the confirmatory sample will also be checked for correlations between variables.

#### Process Validation

The entire research process will be documented in detail to ensure the reliability and transparency of the procedure. This includes data collection, analysis, and interpretation of the data. It also ensures that the questionnaire and the resulting findings fit into existing theoretical frameworks or contribute to the development of new theories. Theory-led validation underpins the relevance and depth of the system.

This study is part of a larger project. The remaining 2 SyMOA dimensions are also planned to be developed into questionnaires.

### Ethical Considerations

This study was approved by Ethikkommission Universitätsklinikum Freiburg on May 9, 2025, under trial ID 25-1117-S2. This study is conducted in accordance with the local legislation and institutional requirements. The participants will provide their written informed consent to take part in this study. All data will be collected via the external online panel provider Bilendi. No personally identifiable information will be collected or stored by the research team, and all responses will be fully anonymized before analysis. As such, participant confidentiality is guaranteed throughout the study. Panel participants will be compensated by Bilendi in accordance with the platform’s standard remuneration procedures.

## Results

As of April 2025, a first draft of 158 items has been developed based on dimension 1 of the SyMOA framework. These items cover constructs such as organizational self-regulation, perception of challenges, modes of communication, and attachment capacity. The draft has undergone an expert review process with 2 OPD-trained experts, who provided feedback on content validity and conceptual alignment. On the basis of this feedback, approximately 20% of the items have been revised to enhance clarity and theoretical precision.

Data collection using the online panel is scheduled for the end of May 2025. Iterative item analyses, including evaluation of item distributions, reliability, and preliminary factor structure, will be conducted following data collection.

## Discussion

### Principal Findings and Comparison to Prior Work

Building on the authors’ previous work, the SyMOA, a questionnaire will be developed. By formalizing systems psychodynamic diagnostics through psychometric means, we seek to enhance organizational self-awareness; improve leadership insight; and contribute to healthier, more emotionally sustainable workplaces. This work addresses a significant gap in the organizational diagnostics landscape and contributes to a broader agenda of integrating psychodynamic psychological insights into evidence-based organizational development.

### Strengths and Limitations

The questionnaire can be used as a screening instrument or as a supplement to comprehensive multimodal diagnostics. In addition, the application of the questionnaire is more user-friendly and economical as, in contrast to the SyMOA interview, no one-on-one setup with a 1-hour time frame is required. Thus, the questionnaire enables a larger number of employees to be surveyed within a shorter time.

This study is firmly grounded in systems psychodynamics theory, translating the new qualitative SyMOA diagnostic tool into a quantitative self-assessment instrument. The multistage design—including expert validation, pilot testing, test-retest reliability, and CFA—reinforces the methodological rigor and psychometric robustness of the resulting questionnaire.

The instrument’s development addresses all key aspects of validity: expert validity, factorial and discriminant validity, and criterion-related (external) validity. This comprehensive approach provides a well-rounded assessment of the questionnaire’s reliability and practical utility.

Importantly, the study fills a well-recognized methodological gap in the field: the absence of empirically validated psychometric tools for systems psychodynamic organizational diagnostics. By offering a scalable, user-friendly questionnaire, this study has high practical relevance for organizational consultants, human resources professionals, and academic researchers alike.

Finally, this study acknowledges the interplay between organizational-level dynamics and intraindividual experiences and, thus, the possible influence of personal perceptions and character traits on collective organizational phenomena. By testing discriminant validity against clinical diagnostic tools (OPD-SF), this study is able to assess this interplay.

### Future Directions

Future research should extend this work by developing and validating questionnaire-based measures for the remaining dimensions (2-3) of the SyMOA framework. This study focuses exclusively on the development and validation of a questionnaire based on dimension 1 of the SyMOA framework, which addresses sociotechnical integration and the internal level of organizational functioning. Accordingly, the validity evidence obtained in this study applies only to this dimension and does not yet support claims regarding the assessment of organizational dynamics in their entirety. Expanding beyond dimension 1 will enable a more comprehensive operationalization of systems psychodynamic organizational diagnostics and allow for an integrated assessment across multiple organizational domains.

A further essential step will be the normative calibration of the instrument. This study is explicitly focused on the development and initial psychometric validation of the SyMOA-Q, including the examination of factorial structure, internal consistency, and first evidence of construct validity. Establishing norms based on data from organizations across different industries, organizational sizes, and hierarchical levels is necessary to enhance the interpretation of scores. In this study, scoring is conceptualized at the level of factor scores for the subscales of dimension 1, with the option of examining higher-order factor structures in subsequent research.

In addition, multilevel research designs represent a key next step. This study is an initial step in the development and psychometric validation of the SyMOA-Q focusing on systems psychodynamic organizational dynamics as perceived by individuals. Future studies should use clustered sampling designs in which multiple respondents are nested within clearly identified organizations. This will allow for the examination of aggregation statistics (eg, intraclass correlations and within-group agreement indexes) to determine whether and under which conditions individual perceptions can be meaningfully aggregated to the organizational level. Building on these analyses, multilevel CFAs should be conducted to disentangle individual- and organization-level variance components and test the factorial structure of the SyMOA dimensions across levels of analysis.

### Dissemination Plan

The results of this study will be disseminated through multiple channels targeting both academic and applied audiences. Scientific dissemination will include publication in peer-reviewed journals focusing on organizational psychology, psychometrics, and systems psychodynamics research, as well as presentations at national and international conferences in these fields.

To support practical implementation, the SyMOA-Q will be introduced to organizational consultants, human resources professionals, and leadership development practitioners through workshops, professional training programs, and practitioner-oriented publications. In addition, a standardized manual including theoretical background, administration guidelines, scoring procedures, normative data, and interpretation frameworks will be developed to ensure consistent and responsible use of the instrument.

All data collected in future studies, including clustered organizational samples for norming, will be pseudonymized to protect individual participants and organizations. Norms and interpretive benchmarks will be shared only at aggregated levels, preventing the identification of specific organizations and ensuring ethical and responsible dissemination.

Further dissemination will involve collaboration with organizations participating in the norming studies, allowing for feedback loops between research and practice. Finally, the instrument may be made available in digital format to facilitate future research collaborations.

## Abbreviations

CFA: confirmatory factor analysis
SyMOA: Systematic Multidimensional Organizational Assessment
SyMOA-Q: Systematic Multidimensional Organizational Assessment–Questionnaire
